# DNA ‘Breathing’ Recombination Cloning: A Mismatch-Tolerant, Temperature-Dependent Homologous Recombination Cloning Method

**DOI:** 10.3390/ijms27062604

**Published:** 2026-03-12

**Authors:** Yun He, Yi Ding, Yan Zhang, Like Liu, Shanhua Lyu, Yinglun Fan

**Affiliations:** College of Agriculture and Life Science, Liaocheng University, Liaocheng 252000, China; 2410190302@stu.lcu.edu.cn (Y.H.); 2510190402@stu.lcu.edu.cn (Y.D.); 2310190207@stu.lcu.edu.cn (Y.Z.); liulike@lcu.edu.cn (L.L.); lvshanhua@lcu.edu.cn (S.L.)

**Keywords:** homologous recombination cloning, melting temperature (TM), PCR product, mismatched bases, DNA ‘breathing’ recombination cloning

## Abstract

DNA cloning traditionally relies on two approaches: restriction endonuclease digestion-ligation, and homologous recombination involving exonucleases, polymerases, and other enzymes. Here, we present a novel cloning method that requires only restriction endonucleases, eliminating the need for exonucleases or polymerases. The linearized cloning vector and the foreign DNA fragment (FDF) containing overlapping sequences were mixed and incubated at the melting temperature of the overlapping DNA sequences for 5 min, then cooled slowly to 0 °C. The mixture was transformed into *E. coli* and positive transformants were obtained. This cloning method was named DNA ‘breathing’ recombination (DBR) cloning. The overlapping sequence between the linearized vector and the FDF is preferably from 12 to 16 base pairs. Even when the ends of the linearized vector contain mismatches of up to 20 base pairs with the ends of the FDF, the DBR cloning method can still proceed efficiently, enabling truly seamless assembly. Meanwhile, the DBR method supports one-step assembly of multiple fragments. Therefore, the DBR cloning method simplifies experimental operations and reduces experimental costs while maintaining high cloning efficiency.

## 1. Introduction

DNA cloning is a foundational molecular biology technology that underpins gene cloning and functional analysis, and also serves as a cornerstone for synthetic biology [1]. The traditional restriction endonuclease digestion–ligase ligation method was the earliest cloning method used [2]. Cloning technologies based on homologous recombination achieve seamless ligation by utilizing the homology of DNA sequences, thus avoiding the limitations of traditional restriction digestion and ligation. Gibson Assembly relies on the synergistic action of three enzymes (including 5′ → 3′ exonuclease, DNA polymerase, and DNA ligase) to complete ligation in vitro without the involvement of host cells in recombination [3,4]. In-Fusion cloning depends on the In-Fusion enzyme (a fusion enzyme with both DNA polymerase and ligase activities) to cleave DNA and generate 5′ single-stranded overhang. Following the annealing of homologous sequences, the enzyme further catalyzes gap repair and phosphodiester bond ligation, ultimately yielding a closed circular recombinant plasmid [5]. Such homologous recombination-based cloning methods have high cloning efficiency, and many biotech companies have produced corresponding kits for promotion and sale in the market, but the prices are high [6]. LIC-PCR (ligation-independent cloning of PCR products) and SLIC (sequence- and ligation-independent cloning) derived from LIC-PCR also rely on T4 DNA polymerase [7,8,9]. Gateway cloning is dependent on the site-specific recombination mechanism of λ phage, which enables the efficient transfer of target genes between different vectors through the mediation of integrase (Int), excisionase (Xis), and integration host factor (IHF) [10,11].

IVC (in vivo cloning) is a homologous recombination-based cloning method in *E. coli* [12,13]. Linearized vectors and PCR amplification products are mixed in vitro and then transformed into *E. coli*, and the cloning of PCR products is completed by utilizing the DNA repair system in *E. coli*, forming a complete plasmid. This method was first published simultaneously by two independent research groups in 1993 [12,13]. After analyzing these reports focusing on homologous recombination through *E. coli*, we found that, in most studies, the mixture of exogenous DNA and vector was treated at room temperature before transformation [12,13,14,15,16]; in contrast, one report performed this treatment of the DNA/vector mixture at 97–100 °C [17]. Therefore, we believe that ‘temperature’ plays a crucial role in homologous recombination through *E. coli*.

Temperature control is critical for DNA hybridization and PCR, as double-stranded DNA denatures into single-stranded DNA under elevated temperatures. During the annealing step of PCR, primers bind to the template strand via base-pairing rules (A-T pairing, G-C pairing) [18]. Upon cooling, single-stranded DNA hybridizes with complementary sequences (homologous DNA or related RNA) through hydrogen bonding, forming stable double-stranded structures [19]. The stability of these duplexes increases as temperature decreases [20]. Double-stranded DNA in organisms is a highly structured and synergistically stable system at physiological temperatures, but, when slightly above physiological temperatures, it is only in a marginally stable state and undergoes a cooperative ‘melting phase transition’ [21]. The length and the sequence heterogeneity of double-stranded DNA make AT-rich fragments more unstable than GC-rich fragments [22]. Therefore, the DNA genome has thermally driven structural ‘breathing’ fluctuations at physiological temperatures [21]. DNA ‘breathing’ refers to the phenomenon where nucleic acid base pairs are temporarily opened due to thermal fluctuations when the experimental temperature is lower than the melting point of double-stranded DNA [23]. DNA ‘breathing’ is a thermally driven process. For the double-stranded DNA at both ends of a short DNA fragment, the hydrogen bonds connecting the DNA double strands will temporarily break and re-form as the ambient temperature changes. From this, we think about how to improve the efficiency of the IVC cloning method. We believe that, when performing IVC cloning, different temperature treatments should be set according to the melting temperature (Tm) value of the length of the overlapping sequence. The IVC cloning method is more similar to the DNA ‘breathing’ caused by temperature changes in the double-stranded DNA at the ends of DNA fragments. Therefore, we renamed the IVC cloning method under temperature changes as ‘DNA breathing recombination’ (DBR) cloning, to facilitate researchers’ understanding and widespread application.

In this study, we established a DBR cloning method based on different temperature treatments according to the Tm value of the overlapping sequence. We investigated the effect of overlapping sequences of various lengths on DBR cloning efficiency, as well as the cloning efficiency when there were mismatched bases at the ends of the FDF fragment and the vector.

## 2. Results

### 2.1. Tm Treatment Significantly Improves DBR Cloning Efficiency

**Figure 1:**
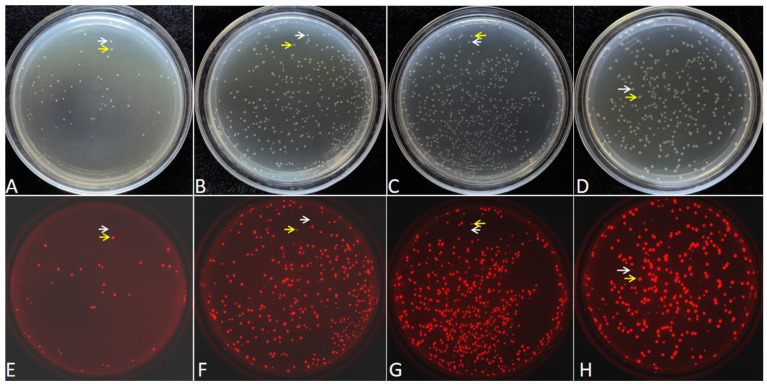
Examples of transformation plates of cloned FDFs harboring the *mScarlet-I* expression cassette. A quarter of the transformed bacterial solution was spread on one LB/Kan plate. (**A**,**E**), (**B**,**F**), (**C**,**G**), and (**D**,**H**) show the transformation recombinants after different temperature treatments at 25 °C, 40 °C, 55 °C, and 65 °C, respectively. (**A**–**D**) are pictured in natural light, and (**E**–**H**) are detected with excitation at 540 nm, emission at 600 nm. The white arrows indicate non-recombinants, and the yellow arrows indicate positive recombinants.

After the four identical replicate DNA mixtures (the L7-18 PCR product and the linearized vector pCAMBIA-1305.1) were treated at four different temperatures, they were transformed into *Escherichia coli* DH5α. The red fluorescent colonies can be observed on the LB plate (Figure 1). The statistical results showed that, under the treatment at 25 °C, the number of red fluorescent colonies was only 140, while, under the treatments where the maximum temperature reached 40 °C, 55 °C and 65 °C, the number of red fluorescent colonies reached 1073, 1955 and 1328, respectively (Supplementary Table S2).

Among these four different temperature treatments, the transformation efficiency is the highest when the maximum treatment temperature is 55 °C. Still, there is no statistically significant difference compared with that at the maximum treatment temperature of 40 °C. The number of positive clones obtained under the maximum temperature of 55 °C is 14.3 times higher than that under the temperature treatment of 25 °C (Supplementary Figure S1). However, when the maximum treatment temperature is 65 °C, the number of positive clones obtained from one transformation decreases to 1328. The Tm values of the two overlapping sequences are both 55.1 °C, which indicates that higher cloning efficiency can be achieved when the maximum treatment temperature is at or slightly lower than the Tm value, while excessively high temperatures will conversely reduce the cloning efficiency.

Ten clones emitting red fluorescence were randomly selected from the transformants of L7-18 for restriction enzyme, *Hin*dIII, digestion identification. The FDF harbored with two *Hin*dIII sites; therefore, the correct ligated vector produced a 782-bp DNA band (Supplementary Figure S2). The digestion results confirmed that the clones emitting red fluorescence were positive clones. The accuracy rate of screening positive clones via red fluorescence was 100%. In this study, a visual red fluorescent expression cassette was used as the FDF for cloning research. After transformation, whether it is a positive transformant clone can be directly determined by the fluorescence display, which significantly improves the identification efficiency.

### 2.2. The Length of the Overlapping Sequence of the Cloning Vector and the Foreign DNA Affects the Cloning Efficiency

**Figure 2:**
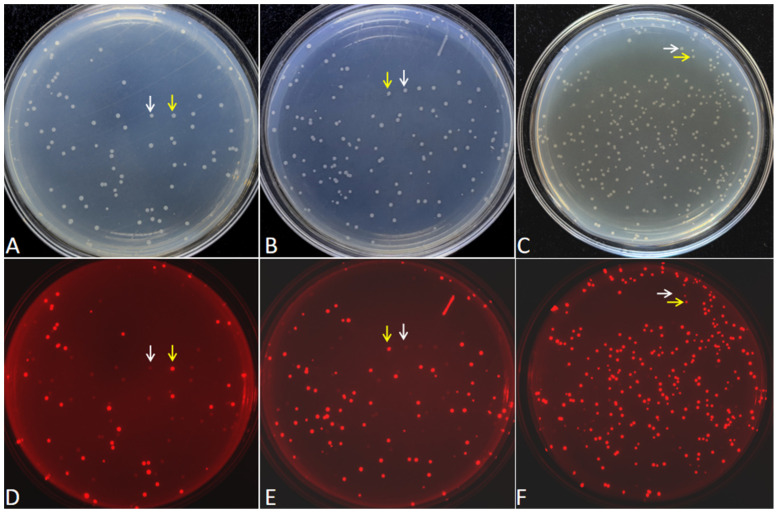
Examples of transformation plates of cloned FDFs harboring the *mScarlet-I* expression cassette. A quarter of the transformed bacterial solution was spread on one LB/Kan plate. (**A**,**D**), (**B**,**E**), and (**C**,**F**) show the transformation recombinants with PCR products harboring with 8, 10, 12 bp length of the overlapping sequence, respectively. Panels (**A**–**C**) show images captured under natural light. Panels (**D**–**F**) show fluorescence images acquired with excitation at 540 nm and emission at 600 nm. The white arrows indicate non-recombinants, and the yellow arrows indicate positive recombinants.

**Figure 3:**
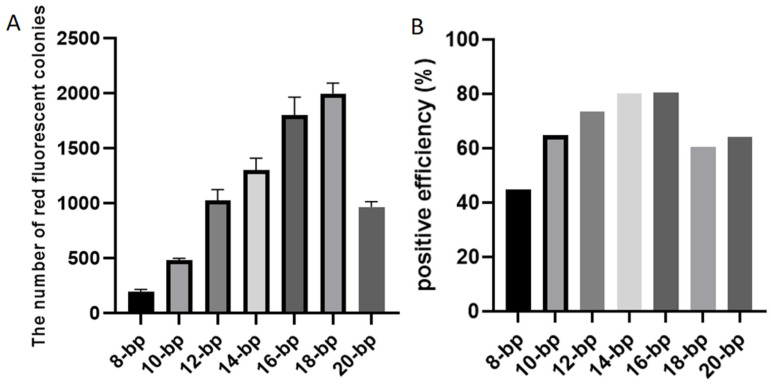
The number of clones emitted red fluorescence (**A**) and the positive efficiency (**B**) with different length of the overlapping sequence. (**A**) Data are the mean ± SD with three biological replicates.

Six FDFs, also each carrying the *mScarlet-I* expression cassette, were PCR-amplified with six primer pairs that introduced overlapping sequences of distinct lengths (8, 10, 12, 14, 16, 18, and 20 bp). To streamline experimental procedures and ensure the uniformity of experimental conditions, a consistent temperature treatment was applied during DBR cloning. The six purified FDFs and the linearized vector pCAMBIA-1305.1 were subjected to a maximum treatment temperature of 55 °C. After transformation, colonies exhibiting red fluorescence indicated successful cloning of the FDFs into the vector pCAMBIA-1305.1. Representative colonies derived from FDFs harboring 8, 10, and 12 bp overlapping sequences with the vector are presented in Figure 2. The colonies formed with the FDFs with 14, 16, 18, and 20 bp overlapping sequences are presented in Supplementary Figure S3. The transformation results demonstrated that the numbers of red fluorescent colonies were 196, 480, 1026, 1301, 1801, 1991, and 967, corresponding with the 8, 10, 12, 14, 16, 18, and 20 bp overlapping sequences, respectively (Supplementary Table S3). As the overlapping sequence extended from 8 bp to 16 bp, the number of red fluorescent colonies gradually increased (Figure 3A). However, when the length of the overlapping sequence increased to 18 and 20 bp, the number of red fluorescent colonies no longer increased (Figure 3A).

To calculate the cloning positive efficiency, the number of colonies emitting red fluorescence and total colonies were counted, respectively. The results showed that, as the length of the overlapping sequence increased from 8 bp to 18 bp, the cloning positive efficiency increased gradually (Figure 3B). The favorable rates for 14 bp and 16 bp overlapping sequences were the same, while the positive rate for the 18 bp overlapping sequence decreased instead (Figure 3B).

The cloning efficiency of the FDF with a 20 bp overlapping sequence was lower than that of the FDF with an 18 bp overlapping sequence at a maximum temperature treatment of 55 °C (Figure 3A). Could this difference be caused by the maximum treatment temperature? We hypothesized that the temperature treatment remained a confounding factor. Consequently, we reprocessed the FDFs with 20 bp overlapping sequences and the vector at a maximum temperature of 65 °C. The results showed that, when the treatment temperature was at 65 °C, the number of positive clone colonies with red fluorescence reached 1360 (Supplementary Figure S4).

### 2.3. Cloning Can Still Be Performed When There Are Base Mismatches Between the Overlapping Sequence of DNA Fragments and Vectors

In both traditional restriction enzyme digestion–ligation cloning and Golden Gate cloning, the sticky ends of the cloning vector generated after enzyme digestion must be entirely complementary and paired with the sticky ends of FDF. In homologous recombination-based cloning methods (e.g., In-Fusion), the overlapping sequences between the vector and FDFs are required to be completely identical. However, in certain research scenarios, it may be necessary to delete or modify specific segments of the vector’s cloning site sequences. In such cases, the overlapping sequences between the vector and the FDF may not be completely identical. Therefore, in this study, we designed FDFs with mismatched bases in their overlapping sequences to investigate whether such FDFs can be cloned using the DBR.

**Figure 4:**
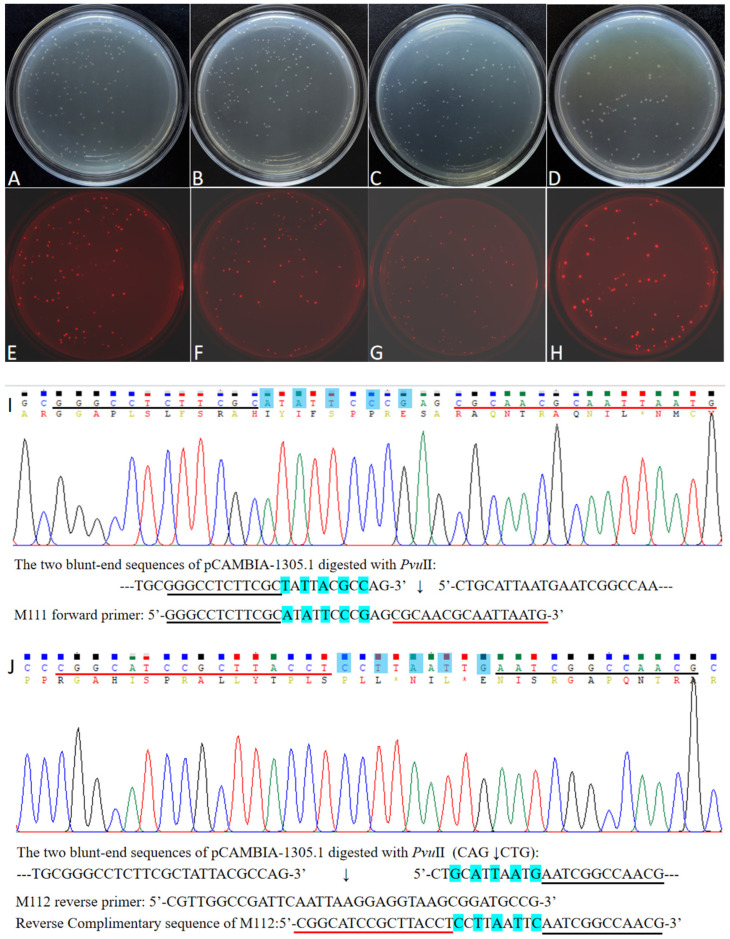
Examples of transformation plates of cloned FDFs with mismatched bases (**A**–**H**) and the Sanger sequencing of M11 positive transformant (**I**,**J**). A quarter of the transformed bacterial solution was spread on one LB/Kan plate. (**A**,**E**), (**B**,**F**), (**C**,**G**), and (**D**,**H**) show the transformation recombinants with M5, M7, M9, and M11, respectively. (**A**–**D**) are pictured in natural light. (**E**–**H**) are detected with excitation at 540 nm, emission at 600 nm. (**I**,**J**) are the Sanger sequencings of M11 with the primer 1305RF and DsRedF, respectively. Notes: The red underlined sequence is the primer that perfectly matches the template for PCR amplification of this fragment. The black underlined sequence is the overlapping sequence with the vector. The bases highlighted in blue correspond to the mismatched nucleotides.

The four FDFs, M5, M7, M9, and M11, were amplified with vector pYFRed as a template and were designed with a 12 bp overlapping sequence starting at the 6th, 8th, 10th, and 12th bases at the end of the PCR product, respectively. The four FDFs and the linearized vector pCAMBIA-1305.1 were subjected to the DBR cloning at a maximum temperature of 55 °C. After transformation, colonies emitting red fluorescence appeared on the LB plate (Figure 4). The numbers of positive colonies were 420, 351, 311, and 209, and the percentage of positive clones was 59.32%, 58.57%, 57.67%, and 53.22%, corresponding with M5, M7, M9, and M11, respectively (Supplementary Table S4). This indicates that the cloning efficiency decreased as the number of mismatched bases increased. When the M11 fragment mismatched at the 3rd, 5th, 7th, 9th, and 11th positions (with the overlapping sequence from position 12 to 23 being identical), the cloning efficiency still reached 209. This indicates that, even if there are some uncomplementary base pairs between the ends of the FDF and the vector, cloning can still be performed. Ten transformants of M11 emitting red fluorescence were randomly selected for digestion with the *Hin*dIII restriction enzyme. The results showed that all ten transformants yielded a 782 bp fragment and indicated that the ten transformants emitting red fluorescence were positive (Supplementary Figure S2). Sanger sequencing of the red fluorescence clones of FDFs M9 and M11 revealed that the sequence of the connection point between FDFs and the vector was entirely identical to the sequence of the designed primers, and the mismatched base pairs were removed from the vector after the cloning was completed (Figure 4I,J and Supplementary Figure S5). So, the DBR cloning method can not only achieve seamless ligation but also allows modification of the DNA sequence at the end of the vector.

During the cloning process of the M11 fragment, the 11 bp sequence at the end of the linearized vector was mismatched with the PCR product. Therefore, it was necessary to denature the 23 bp sequence at the end of the linearized vector to expose the 12 bp overlapping region homologous to the PCR product. Therefore, the M11 fragment and the linearized vector pCAMBIA-1305.1 were subjected to temperature treatment at a maximum temperature of 70 °C. The results demonstrated that the number of positive clones obtained at 70 °C reached 336, representing a significant 50% enhancement in cloning efficiency compared with the 55 °C treatment (Supplementary Figure S6).

**Figure 5:**
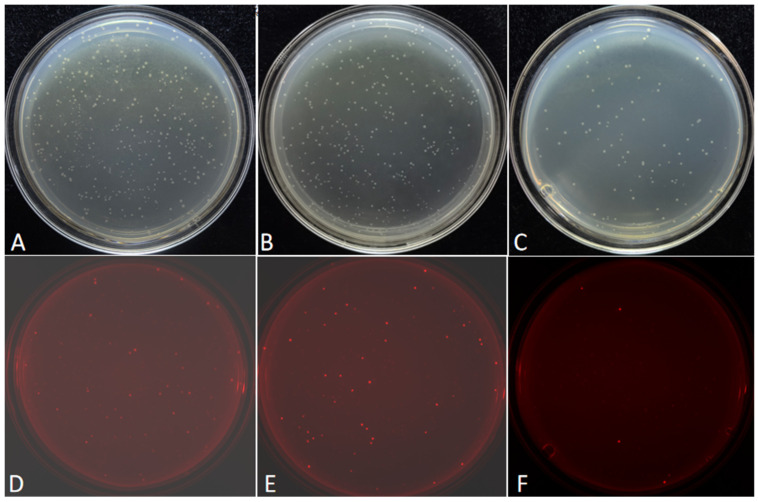
Examples of transformation plates of cloned FDFs with mismatched bases (**A**–**F**). (**A**,**D**), (**B**,**E**), and (**C**,**F**) show the transformation recombinants with M17, M20, and M25, respectively. (**A**–**C**) are captured under natural light. (**D**–**F**) are acquired with excitation at 540 nm and emission at 600 nm.

**Figure 6:**
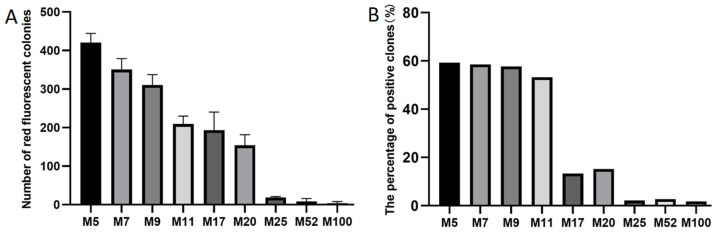
The number of clones emitting red fluorescence (**A**) and the percentage of positive clones (**B**) achieved by the DBR cloning method. M5, M7, M9, M11, M17, M20, M25, M52, and M100 are the different PCR products with different lengths of mismatched base pairs, 5 bp, 7 bp, 9 bp, 11 bp,17 bp, 20bp, 25 bp, 52 bp, and 100 bp sequences at the linear vector ends, respectively.

**Figure 7:**
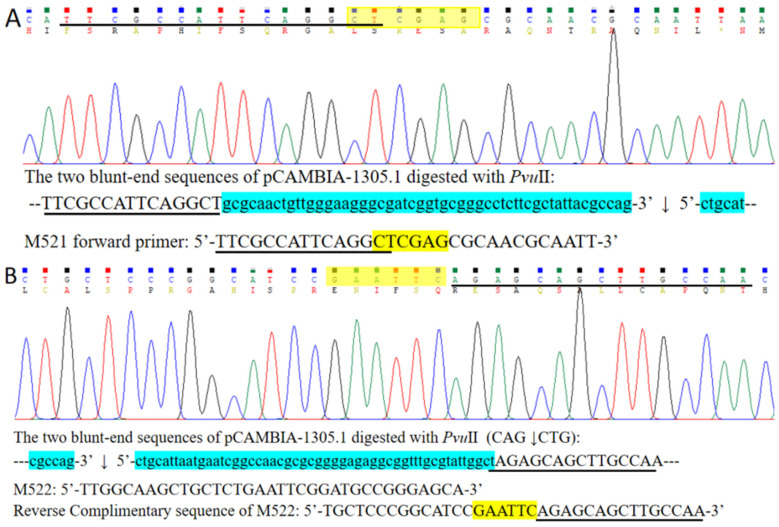
The Sanger sequencing of M52 with the primers 1305RF (**A**) and DsRedF (**B**). Notes: The black underlined sequence is the overlapping sequence with the vector. The blue-colored highlighted bases are the mismatched bases. The sequences highlighted with yellow color are the restriction sites added.

Through the cloning of the aforementioned M5, M7, M9, and M11 fragments, it is noteworthy that the DBR cloning method can tolerate an 11 bp mismatch while still achieving relatively high cloning efficiency. Then, what is the maximum length of base mismatch that the DBR cloning method can tolerate? Another five FDFs were amplified with the five primer sets M171/M171, M201/M201, M251/M252, M521/M522, and M100F/M522 and were named with M) as M17, M20, M25, M52, and M100, respectively. Red fluorescent positive clones were observable on LB plates inoculated with the transformation products (Figure 5 and Supplementary Figure S7). However, as the number of mismatched bases increased, both the cloning efficiency and the transformant positive rate exhibited a significant decrease (Figure 5 and Figure 6 and Supplementary Table S5). The Sanger sequencing to the red fluorescent positive clone of M52 showed that the sequence was identical to the amplified sequence of the designed primers M251/M252, and all mismatched sequences at the vector ends were eliminated (Figure 7). However, when the length of mismatched bases exceeded 25 bp, both the cloning efficiency and the positive rate decreased sharply (Figure 6). Six and seven clones emitting red fluorescent were selected from the transformants of M25 and M52 respectively. Restriction enzyme digestion with *Xho*I showed that five clones from each group had correct digestion results (Supplementary Figure S8). This indicates that the accuracy of restriction enzyme digestion-based detection is less than 100%, suggesting that, as the number of mismatched bases increases, the probability of erroneous recombination during annealing is elevated.

### 2.4. Successful Multi-Fragment Assembly Based on DBR

**Figure 8:**
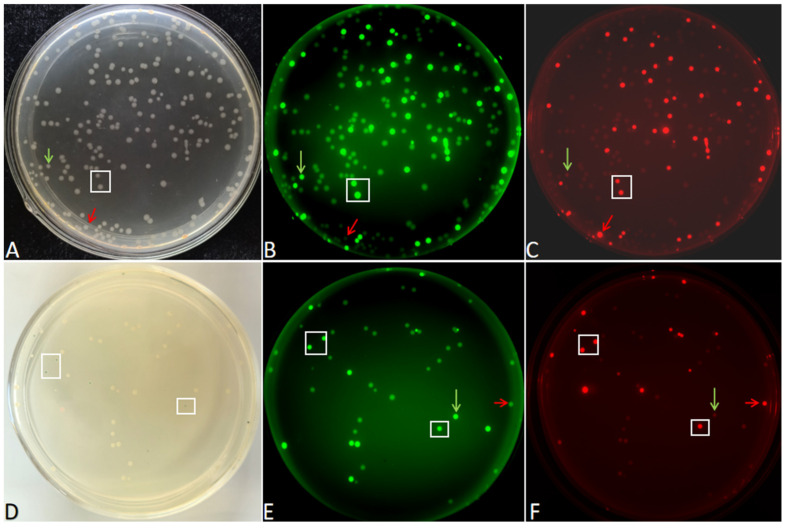
Two (**A**–**C**) and three (**D**–**F**) FDFs assembled by DBR. The two colonies in the white boxes (**A**–**C**) emitted both green and red fluorescence simultaneously. The two colonies in the white boxes (**D**–**F**) showed blue color and emitted both green and red fluorescence simultaneously. The green arrows: the colony only emits green fluorescence but not red fluorescence. The red arrows: the colony only emits red fluorescence but not green fluorescence.

To further demonstrate that DBR can also assemble multiple FDFs, two FDFs, the green fluorescent and red fluorescent gene expression cassettes, were amplified for DBR cloning. There were three 15 bp overlapping homologous arms between adjacent fragments in the two FDFs and the linearized vector pCAMBIA-1305.1. After *E. coli* transformation, colonies emitting both green and red fluorescence were observed on the LB plate (Figure 8A–C). One transformation yielded 276 green fluorescent clones and 256 red fluorescent clones, among which 240 clones emitted both green and red fluorescence simultaneously. This indicates that these 240 clones completed the assembly of the two FDFs.

Subsequently, three FDFs were amplified for DBR cloning. In addition to the green fluorescent and red fluorescent gene expression cassettes, one more FDF was added, which was the *LacZα* fragment from the vector pUC19. These three FDFs and the linearized vector pCAMBIA-1305.1 were used with DBR cloning and transformed into *E.coli* DH5α. There were some colonies that showed a blue color and emitted both green and red fluorescence on the LB/IPTG/X-gal plates (Figure 8D–F). Six positive clones were randomly selected, and plasmid DNA was extracted from each. Restriction enzyme digestion with *Xho*I showed that these two FDFs had been cloned into the vector pCAMBIA-1305.1 (Supplementary Figure S9). This indicates that these three FDFs were cloned into the vector pCAMBIA-1305.1. However, the cloning efficiency was relatively lower than that of two FDFs cloning, and only 16 positive clones were obtained in one transformation.

## 3. Discussion

When cloning exogenous DNA, the molar ratio of FDF to vector is a critical parameter. Optimizing this ratio maximizes productive end-to-end collisions and, consequently, the yield of recombinant plasmids. To out-compete side reactions such as vector re-ligation, concatemer formation, plasmid dimers, or multi-insert plasmids, insert molecules must be present in excess, hence the higher molar amount relative to the vector [24]. For a traditional restriction-ligation cloning method with cohesive ends, the FDF to vector ratio of 1–3:1 is recommended [25]. The 2:1 molar ratio is specified in both In-Fusion cloning and IVC cloning [5,12]. Following these previous reports [5,12,16], we adopted a 2:1 ratio without further optimization.

The efficiency of homologous recombination in *E. coli* is still relatively low in the reported studies. For example, in the optimized ligation system (with the optimal amount of insert and vector at a molar ratio of 2:1 and 30 bp overlapping sequences), only about 120 positive clones per transformation (50 μL DH5α competent cells) were obtained when the PCR product was cloned into the pUC19 vector [14]. In this study, the different FDFs and the linearized vector were treated with varying treatments of temperature and then transformed into *E. coli*. The results showed that when the treatment temperature was equal to or higher than the Tm of the overlapping sequence, the cloning efficiency was significantly improved. When the FDF carried an 18 bp overlapping sequence, the number of positive clones obtained per transformation reached 2000 after treatment at 55 °C. The cloning efficiency of DBR cloning was already comparable to that used in gene library construction [26]. Thus, it can be concluded that pre-treating the FDF and the linearized vector at a temperature equivalent to the Tm of the overlapping sequence before transformation significantly enhances cloning efficiency. Based on basic biological knowledge, the Tm of DNA refers to the temperature at which 50% of double-stranded DNA is denatured into single strands during heating. Essentially, Tm reflects the difficulty of breaking the hydrogen bonds between the two strands of double-stranded DNA. The higher the Tm, the more stable the double-stranded DNA and the higher the temperature required to denature it. When double-stranded DNA is near its denaturation temperature, RNA-DNA hybrids exhibit higher thermodynamic stability [27]. In a low-temperature environment, the collision probability between single strands decreases, the mismatch probability increases, and the local structure formed by mismatches is stabilized through hydrogen bonds, creating an irreversible kinetic trap [28]. These studies above can explain why the efficiency of the homologous recombination-based cloning method is relatively low at room temperature. Integrating the findings of this study, we conclude that high cloning efficiency can be achieved when the maximum treatment temperature approaches the Tm. During DNA complementary pairing, GC pairs are linked by three hydrogen bonds, whereas AT pairs are linked by two. Theoretically, a high GC content may improve cloning efficiency. However, since the overlapping sequences were relatively short, we did not observe that overlapping sequences with high GC content significantly increased cloning efficiency in our experiments. In most cases, there is limited choice for selecting overlapping sequences during DNA cloning. Therefore, it is not necessary to consider the GC content of the overlapping sequences; FDFs and the linearized vector can be performed simply according to the Tm value of the overlapping sequences.

Early studies have shown that the efficiency of DNA renaturation (annealing) is highly dependent on the rate of temperature change, and slow cooling is the key to ensuring that complementary strands fully collide through Brownian motion and form correct pairings [29]. As is well established in molecular biology, after double-stranded DNA denatures into single-stranded DNA at elevated temperatures, rapid cooling of the reaction environment prevents the single-stranded DNA from annealing to re-form double-stranded DNA within a short timeframe. This is because rapid cooling prevents the ssDNA strands from finding their correct complementary sequences through Brownian motion, resulting in the formation of locally mismatched ‘frozen’ structures [30]. The core mechanism underlying the failure of renaturation following double-stranded DNA denaturation upon rapid cooling stems from the combined effects of kinetic limitations and thermodynamic traps. When the cooling rate exceeds 5 °C/s, the DNA renaturation yield drops sharply from 90% to less than 10% [28,31]. The renaturation rate of fully denatured DNA follows second-order reaction kinetics. When the temperature drops below the Tm, the reaction rate accelerates, and then decreases as the temperature further lowers [32,33]. Therefore, during temperature treatment of the vector and the FDF mixture, following incubation at the Tm for 5 min, we applied a slow-cooling protocol (decreasing by 5 °C every 3 min) to provide sufficient time for the overlapping sequences to renature.

We referred to the homology arm lengths of other cloning methods based on homologous recombination (e.g., the homology arm lengths corresponding to Gibson Assembly, In-fusion, and SLIC are 15–40 bp, 15–25 bp, and 30–60 bp, respectively) [3,5,8]. In this study, the overlapping sequences of varying lengths were appended to the 5′ ends of the primers, enabling the amplified PCR fragments to share identical homologous sequences with the termini of the cloning vector. A total of seven overlapping sequences with different lengths (ranging from 8 bp to 20 bp) were designed. These amplification products and the linearized vector were transformed into *E. coli* after temperature-controlled treatment. The results showed that as the length of the overlapping sequence increased from 8 bp to 18 bp, both the number of positive transformants per transformation and the proportion of positive transformants gradually increased. At higher temperatures, the dsDNA at the vector ends and FDF ends unwinds into ssDNA, and as the temperature decreases, the ssDNA at the vector ends and the ssDNA at the FDF ends have the opportunity to re-base pair and form hydrogen bonds, resulting in the formation of short dsDNA and further leading to the formation of four-way DNA structures (Holliday junction) [34,35]. Therefore, the longer the overlapping sequence, the more stable the formed double-stranded DNA structures (or four-way DNA structures), which leads to the gradual increase in both the number of positive transformants and the proportion of positive transformants. However, it does not mean that the longer the overlapping sequence the better. In the cloning of FDFs with lengths from 8 bp to 20 bp in this study, the cloning efficiency of the 18 bp FDF was the highest. Even when the treatment temperature was increased, the cloning efficiency of the 20 bp overlapping sequence was significantly lower than that of the 18 bp one. This could be due to the increase in the length of overlapping sequences, which leads to a rise in mismatches, thereby resulting in a decrease in cloning efficiency. Considering both transformation efficiency and cost, we recommend that the length of the overlapping sequence be 14–16 bp.

The efficiency of homologous recombination cloning methods, such as Gibson Assembly, In-Fusion cloning, and LIC/SLIC, depends critically on complete sequence homology between the vector and FDF ends. Mismatches in overlapping sequences significantly reduce recombination efficiency or even lead to experimental failure by interfering with the synergistic action of exonucleases, polymerases, and ligases [3,8]. In this study, we designed mismatched sequences in the PCR products compared with the ends of the digested vector. The results showed that, even when four spaced mismatched bases were introduced into the M9 PCR product, the FDF could still be successfully cloned using the DBR cloning method with relatively high efficiency, yielding approximately 310 positive clones per transformation with a positive clone rate of 57.67%. When the number of mismatched bases reached 20 bp, approximately 154 positive clones were obtained per transformation, and the positive clone rate decreased to 15.2%. When the number of mismatched bases exceeded 25 bp, the number of positive clones dropped sharply, and the positive clone rate decreased to 2%. This may be because when the number of mismatched bases exceeds 25 bp, the overlapping sequences can only pair with each other after at least 40 bp of the vector ends are unwound. However, unwinding 40 bp or 52 bp of the vector ends requires increasing the treatment temperature to above 84 °C or 93 °C, at 93 °C all double-stranded DNA is likely to be completely unwound. During the subsequent cooling process, the probability of mismatching in double-stranded DNA increases significantly, thereby reducing the cloning efficiency. Sequencing results of positive recombinants confirmed that the sequences of positive clones after recombination were identical to that of FDF. This implies that when FDF and the vector were mixed and transformed into *E. coli*, the mismatched sequence segment at the vector ends was eliminated during the repair process in *E. coli*. In the presence of mismatched bases, these mismatched sequences cannot form a Holliday junction; instead, they exist as single-stranded segments outside the Holliday junction, forming a tail of the Holliday junction. During the repair process in *E. coli*, these single-stranded sequences, along with part of the complementary sequences, are excised. Therefore, the DBR cloning method affords greater cloning flexibility, and tailored primers can precisely modify sequences adjacent to restriction sites, achieving truly seamless assembly. The tolerance of the DBR cloning method to mismatches in overlapping sequences breaks the technical limitation of ‘complete homology dependence’, providing crucial support for enhancing the flexibility, efficiency, and application scope of molecular cloning.

In this study, the cloning of multiple FDFs could also be accomplished using the DBR cloning method. However, the cloning efficiency of multiple FDFs was lower than that of a single FDF. Meanwhile, the efficiency of cloning two FDFs was 15 times that of cloning three FDFs in this study. In all reported studies, during the ligation of multiple FDFs, the cloning efficiency gradually decreased as the number of fragments increased [5,16,17]. This indicates that there is indeed an issue of low cloning efficiency for multiple FDFs. The possible reasons for the low efficiency of multiple FDFs cloning are as follows: multi-fragment cloning poses two major challenges—precise control of equimolar ratios among multiple fragments is difficult to achieve, and an increased number of fragment ends elevates the risk of end-to-end misannealing—thereby compromising cloning efficiency.

VIC cloning efficiency in E. coli is largely dependent on intramolecular gap repair, which represents the primary DNA repair pathway documented in existing studies [12,14]. However, we disagree with this view. We believe that DBR cloning (or VIC cloning) occurs because the homologous ends of the dsDNA of FDF and the vector form relatively stable Holliday junctions during annealing [34,35]. FDF and the vector form a circular plasmid through two Holliday junctions; after this, the circular plasmid is transformed into *E. coli*, and the repair system in *E. coli* repairs the Holliday junctions into dsDNA. Studies have shown that cells have evolved multiple pathways to ensure the timely removal of Holliday junctions. The bacterial protein RuvC is a canonical resolvase that introduces two symmetric cuts in the Holliday junction. For complete resolution of the HJ, the two cuts need to be tightly coordinated [35]. One of these pathways involves the nuclease processing of Holliday junctions by special structure-specific endonucleases called ‘resolvases’, which cut the connections between linked molecules [36,37].

## 4. Materials and Methods

### 4.1. Preparation of Linearized Vector

The cloning vector used in this study is the Binary vector pCAMBIA-1305.1 (GenBank No. AF354045.1, Supplementary Figure S10). The method for preparing linearized vector is as follows: 1 μg plasmid DNA digested with 200 U of *Pvu*II in 1× cutsmart buffer (NEB Biotech, Ipswich, MA, USA) for 2 h. Then, add 2.5 times the volume of absolute ethanol, centrifuge at 12,000 rpm for 15 min, discard the alcohol, air-dry at room temperature, dissolve with 100 μL of 1×TE, and the concentration of the linearized vector pCAMBIA-1305.1 is approximately 50 ng/μL.

### 4.2. Foreign DNA Fragments, PCR Amplification, and Transformation

In this study, a total of three FDFs were selected for DBR cloning. FDF 1 (FDf-1) is the red fluorescent gene expression cassette, which is amplified from the vector pYFRed *(mScarlet-I*, driven by the Lac promoter, GenBank No. PQ778476) [38] or pMRE-Tn5-145 (*mScarlet-I*, driven by the Npt2 promoter, Addgene plasmid No. 118529) [39]. FDf-2 is the expression cassette of *sGFP2* gene driven by the *NptII* promoter form the plasmid pMRE-Tn5-152 (Addgene plasmid No. 118534) [39]. FDf-2 was used in two/three foreign fragments joined. The positive recombinants of FDf-1 and FDf-2 with the vector will enable *E. coli* to exhibit both red and green fluorescence.

FDFs were amplified with KOD One PCR Master Mix, blue (TOYOBO, Kita-ku, Osaka, Japan) with 0.5 μL forward and 0.5 μL reverse primers (10 nmol/L) and 0.5ng plasmid DNA with a total of 25 μL. The PCR products were purified using a SanPrep Column PCR Product Purification Kit (Sangon Biotech (Shanghai) Co., Ltd., Shanghai, China) according to the manufacturer’s instructions.

The mixture of FDFs and the linearized vector pCAMBIA-1305.1 were transformed with 100 μL DH5α competent cells (CaCl_2_ treatment, transformation efficiency more than 10^7^ cfu/μg DNA), and heat shocked at 42 °C for 45 s. Subsequently, 500 μL antibiotic-free LB medium was added, and the transformed bacteria solution was incubated at 37 °C with shaking at 180 rpm for 60 min. Lastly, a quarter of (150 μL) transformed bacteria solution was spread on solid LB medium with 50 mg/L kanamycin.

### 4.3. Effects of Temperature Variations on DBR Cloning Efficiency

Among different homologous cloning methods, the recommended overlapping sequence lengths for Gibson Assembly, In-fusion, and SLIC are 15–40 bp, 15–25 bp, and 30–60 bp, respectively [3,5,8]. Therefore, in this study, PCR products with an 18 bp overlapping sequence length were selected to verify the cloning efficiency under different temperature treatments. Two 18 bp overlapping sequences added to the 5′ end of the primer pair L7-18F/L7-18R were 5′-TCTTCGCTATTACGCCAG-3′ and 5′-GCCGATTCATTAATGCAG-3′ (the overlapping sequences in the primers are marked with underlines in Supplementary Table S1). The Tm values of the overlapping sequences in L7-18F and L7-18R were both 55.1 °C. The PCR product, L7-18, carrying the mScarlet-I expression cassette, was amplified with the primer pair L7-18F/L7-18R (all the primers used in this study are listed in Supplementary Table S1), adding two 18 bp overlapping sequences at the 5′ end of primers, using the cloning vector pYFRed plasmid DNA as a template [38], and the expected size of the PCR product was 1076 bp. In a 0.2 μL centrifuge tube, 6 μL of ultrapure water was added, followed by 1.0 μL of the purified PCR product (10 ng) and 1.0 μL of the linearized vector pCAMBIA-1305.1 (50 ng), making a total system volume of 8 μL, which was then mixed thoroughly The same four replicates of the above mixed samples were prepared, and then subjected to the following temperature treatments: (1) Maintained at 25 °C for 15 min. (2) Placed in a PCR instrument and run with the following program: 40 °C for 5 min, 35 °C for 3 min, 30 °C for 3 min, 25 °C for 3 min, 16 °C for 5 min, 4 °C for 5 min. (3) Placed in a PCR instrument and run with the following program: 55 °C for 5 min, 50 °C for 3 min, 45 °C for 3 min, 40 °C for 3 min, 35 °C for 3 min, 30 °C for 3 min, 25 °C for 3 min, 16 °C for 5 min, 4 °C for 5 min. (4) Placed in a PCR instrument and run with the following program: 65 °C for 5 min, 60 °C for 3 min, 55 °C for 3 min, 50 °C for 3 min, 45 °C for 3 min, 40 °C for 3 min, 35 °C for 3 min, 30 °C for 3 min, 25 °C for 3 min, 16 °C for 5 min, 4 °C for 5 min. After the above temperature controls were completed, the four samples were immediately placed in an ice-water mixture for 5 min and then subjected to transformation. All experiments were conducted with three independent biological replicates to ensure reproducibility.

Six clones emitting red fluorescence were randomly selected to extract DNA and then restriction digestion was performed with *Xho*I to verify whether FDF was inserted into the vector.

### 4.4. Effects of the Overlapping Sequence Length on DBR Cloning Efficiency

The recommended length of overlapping sequence with the cloning vector in the In-Fusion cloning system is 15 bp [5]. Therefore, we used this length as a reference and designed different lengths of overlapping sequences to analyze the cloning efficiency. In this study, a total of 6 overlaps with different lengths (8, 10, 12, 14, 16, and 18 bp) were set. After adding overlapping to the 5′ end of the primers, FDf-1 harboring with *mScarlet-I* was amplified with the fluorescent vector pYFRed as a template. The PCR primers with 6 different overlaps are listed in Supplementary Table S1. PCR products were purified with a SanPrep Column PCR Product Purification Kit (Sangon Biotech (Shanghai) Co., Ltd.) and the purified DNA was dissolved with elution buffer (2.5 mM Tris-HCl, pH 8.5).

A total of 10 ng of the purified PCR product was mixed with the linearized vector pCAMBIA-1305.1 (50 ng), and ultrapure water was added to the total volume of 10 μL. The mixture was subjected to a PCR program as follows: 55 °C for 5 min, 50 °C for 3 min, 45 °C for 3 min, 40 °C for 3 min, 35 °C for 3 min, 30 °C for 3 min, 25 °C for 5 min, 16 °C for 5 min, 4 °C for 5 min, then transferred to ice-water mixture for 5 min and subjected to transformation. The above heat treatment procedure is named DNA breathing annealing (DBA). Then, the mixture was mixed with 100 μL DH5α competent cells in an ice-water bath and heat-shocked at 42 °C for 45 s. Subsequently, 500 μL antibiotic-free LB medium was added, and the mixture was incubated at 37 °C with shaking at 180 rpm for 60 min. A total of 100 μL of the bacterial mixture was spread onto LB harboring with 50mg/L kanamycin, which was incubated in a 37 °C incubator for 16 h. Ten transformants of M11 emitting red fluorescence were randomly selected for digestion with *Hin*dIII restriction enzyme. The results showed that all ten transformants yielded a 782 bp fragment and indicated that the ten transformants emitting red fluorescence were positive (Supplementary Figure S2). The number of total E. coli colonies and colonies showed red fluorescent were counted for cloning efficiency and positive rate. This experiment was performed with 3 independent biological replicates.

### 4.5. Cloning Compatibility of Mismatched Overlapping Sequences

It is unclear whether the DBR cloning method can be performed when there are mismatched bases in the overlapping sequences between foreign DNA and the vector. Since the sequence of the vector used cannot be altered, mismatched bases can be introduced into the designed primers by changing base G to C, or substituting A with T. Based on the overlapping sequences at both ends of the vector cloning site, mismatched bases were introduced into the primers while still maintaining a 12 bp overlapping sequence between the PCR products and the linearized vector. The designed primers and mismatched bases are shown in Supplementary Table S1. The four PCR products were amplified with the four primer sets, M501/M502 (the mismatched bases located at 3rd/5th base positions), M701/M702 (the mismatched bases located at 3rd/5th/7th base positions), M901/M902 (the mismatched bases located at 3rd/5th/7th/9th base positions), and M111/M112 (the mismatched bases located at 3rd/5th/7th/9th/11th base positions), using the plasmid DNA of the vector pYFRed as template. The four purified FDFs were mixed with the linearized vector pCAMBIA-1305.1. Then they were treated using the DBR cloning method with the following PCR program: 60 °C for 5 min, 55 °C for 3 min, 50 °C for 3 min, 45 °C for 3 min, 40 °C for 3 min, 35 °C for 3 min, 30 °C for 3 min, 25 °C for 5 min, 16 °C for 5 min, 4 °C for 5 min, then they were transferred to the ice-water mixture for 5 min and subjected to transformation.

Since the PCR product of M111/M112 has 11 bp mismatched bases with the ends of the vector, if the 12 bp overlapping sequence in the M111/M112 PCR product and the overlapping sequence of the vector form Holliday junctions, it is necessary to unwind the 23 bp double-stranded ends of the vector to enable the complementary pairing of this 12 bp overlapping sequence. The Tm values of the 23 bp ends of the vector were 69.8 °C and 65.8 °C. So the PCR product of M111/M112 and the linearized vector pCAMBIA-1305.1 were treated using the DBR cloning method with the following PCR program: 70 °C for 5 min, 65 °C for 3 min, 60 °C for 3 min, 55 °C for 3 min, 50 °C for 3 min, 45 °C for 3 min, 40 °C for 3 min, 35 °C for 3 min, 30 °C for 3 min, 25 °C for 5 min, 16 °C for 5 min, 4 °C for 5 min, then they were transferred to the ice-water mixture for 5 min and subjected to transformation.

Based on the cloning results of the four aforementioned PCR products harboring mismatched bases, all four fragments were successfully cloned into the linearized vector pCAMBIA-1305.1 via DBR cloning. This finding demonstrates that the DBR cloning method exhibits cloning compatibility even in the presence of mismatched overlapping sequences. Therefore, we planned to extend the mismatched sequences further. A total of five pairs of PCR primers (M171/M171, M201/M201, M251/M252, M521/M522, and M100F/M522) were designed to amplify the mScarlet-I expression cassette. The 12 bp or 15 bp sequences at the ends of these five groups of PCR amplicons were completely homologous to the sequences starting from the 18th, 21st, 26th, 53rd, and 101st positions of the ends of the linearized vector pCAMBIA-1305.1 digested with *Pvu*II. In other words, the 17 bp, 20 bp, 25 bp, 52 bp, and 100 bp sequences at the vector ends shared no homology with the PCR amplicons and were all mismatched sequences. The five FDFs were amplified with the vector pYFRed as the template and purified with the SanPrep Column PCR Product Purification Kit. The five purified FDFs were mixed with the linearized vector pCAMBIA-1305.1, and then treated using the DBR cloning method with the highest temperature, 81 °C, 84 °C, 88 °C, 94 °C and 94 °C, for 5 min, then decreasing 5 °C every 3 min to 4 °C for 5 min, then transferred to an ice-water mixture for 5 min and subjected to transformation. Some transformants emitting red fluorescence were selected for restriction endonuclease digestion verification with *Xh*oI.

### 4.6. Multi-Fragment Assembly by DBR

To further verify the ability and efficiency of the DBR cloning method to clone multiple exogenous DNA fragments, two FDFs were tried to be cloned into the cloning vector. The green fluorescent expression cassette was obtained by amplification with 2Frag1F/2Frag1R and pMRE-Tn5-152 as a template, and the red fluorescent expression cassette was obtained by amplification with 2Frag2F/2Frag23R. There were three 15 bp overlapping sequences between each of these two PCR amplification products and the linearized vector. About 10 ng of each of the two purified PCR products were mixed with 50 ng of the linearized vector pCAMBIA-1305.1 and subjected to DBA treatment. Then, the mixture was transformed into 100 μL DH5α competent cells. The number of total E. coli colonies and colonies emitting both red fluorescent and green fluorescent were counted for cloning efficiency and positive rate.

Further, three FDFs, including the green fluorescent expression cassette, the red fluorescent expression cassette, and the LacZα fragment, were amplified to clone into the cloning vector. The green fluorescent expression cassette was also amplified with primer set 2Frag1F/2Frag1R and pMRE-Tn5-152 as a template. The LacZα fragment was amplified with primer set 3Frg31/3Frg32 and pUC19 as a template. The red fluorescent expression cassette was amplified with 3Frg33/2Frag23R. There were also four 15 bp overlapping sequences in the three PCR amplification products and the linearized vector. The remaining operation steps were the same as the above steps.

## 5. Conclusions

The DBR cloning method established in this study enables efficient molecular cloning by performing temperature treatment based on the Tm value of the overlapping sequence between the vector and the FDF fragment. Meanwhile, it allows base mismatches with long sequences (more than 100 bp) at the ends of the vector and the FDF fragment, breaking through the technical limitation of ‘complete homology dependence’. The DBR cloning method does not need more long overlapping sequences and expensive commercial kits and exonucleases, ligases, polymerases, etc. So this method can significantly reduce experimental costs. Given its simplicity and high efficiency, the DBR cloning method holds great potential to become a routine procedure in most molecular biology laboratories.

## Data Availability

The original contributions presented in this study are included in the article/Supplementary Materials. Further inquiries can be directed to the corresponding author.

## Supplementary Materials

The following supporting information can be downloaded at: https://www.mdpi.com/article/10.3390/ijms27062604/s1.
