# Supplementary material for: DNA ‘Breathing’ Recombination Cloning: A Mismatch-Tolerant, Temperature-Dependent Homologous Recombination Cloning Method
Source: Int J Mol Sci. 2026 Mar 12;27(6):2604. doi: 10.3390/ijms27062604 (PMC13026567; doi:10.3390/ijms27062604)
Supplement: Supplementary file 1 [file ijms-27-02604-s001.zip › ijms-4164534-supplementary.pdf]

Supplementary Table S1. All primers used in this study.

Primers	Sequence (5'-3') Underlined nucleotides were the overlapping sequences with the vector or adjacent fragment to be joined. Base mismatches were highlighted with blue color. The sequences highlighted with yellow color is the restriction sites.	Used in
L7-8F	TACGCCAGCAACGCAATTAATGTGAGT	To amplify *mScarlet-I* with an overlapping sequence of 8-bp
L7-8R	TAATGCAGGTAAGCGGATGCCGGG	
L7-10F	ATTACGCCAGCAACGCAATTAATGTGAGT	To amplify *mScarlet-I* with an overlapping sequence of 10-bp
L7-10R	ATTAATGCAGGTAAGCGGATGCCGGG	
L7-12F	CTATTACGCCAGCAACGCAATTAATGTGAGT	To amplify *mScarlet-I* with an overlapping sequence of 12-bp
L7-12R	TCATTAATGCAGGTAAGCGGATGCCGGG	
L7-14F	CGCTATTACGCCAGCAACGCAATTAATGTGAGT	To amplify *mScarlet-I* with an overlapping sequence of 14-bp
L7-14R	ATTCATTAATGCAGGTAAGCGGATGCCGGG	
L7-16F	TTCGCTATTACGCCAGCAACGCAATTAATGTGAGT	To amplify *mScarlet-I* with an overlapping sequence of 16-bp
L7-16R	CGATTCATTAATGCAGGTAAGCGGATGCCGGG	
L7-18F	TCTTCGCTATTACGCCAGCAACGCAATTAATGTGAGT	To amplify *mScarlet-I* with an overlapping sequence of 18-bp
L7-18R	GCCGATTCATTAATGCAGGTAAGCGGATGCCGGG	
L7-20F	CCTCTTCGCTATTACGCCAGCAACGCAATTAATGTGAGT	To amplify *mScarlet-I* with an overlapping sequence of 20-b
L7-20R	TGGCCGATTCATTAATGCAGGTAAGCGGATGCCGGG	
M301	TCGCTATTACGCGAGCGCAACGCAATTAATG	To amplify *mScarlet-I* with an overlapping sequence of 12-bp and 1-bp mismatche
M302	GATTCATTAATGGAGGTAAGCGGATGCCG	
M501	CTTCGCTATTACCCGAGCGCAACGCAATTAATG	To amplify *mScarlet-I* with an overlapping sequence of 12-bp and 2-bp mismatches
M502	CCGATTCATTAAAGGAGGTAAGCGGATGCCG	
M701	CTCTTCGCTATTTCCCGAGCGCAACGCAATTAATG	To amplify *mScarlet-I* with an overlapping sequence of 12-bp and 3-bp mismatches
M702	GGCCGATTCATTTAAGGAGGTAAGCGGATGCCG	
M901	GCCTCTTCGCTAATTCCCGAGCGCAACGCAATTAATG	To amplify *mScarlet-I* with an overlapping sequence of 12-bp and 4-bp mismatches
M902	TTGGCCGATTCAATTAAGGAGGTAAGCGGATGCCG	
M111	GGGCCTCTTCGCATATTCCCGAGCGCAACGCAATTAATG	To amplify *mScarlet-I* with an overlapping sequence of 12-bp and 6-bp mismatches
M112	CGTTGGCCGATTGTATTAAGGAGGTAAGCGGATGCCG	
M171	CGGTGCGGGCCTGTGAGCGCAACGCAATT	To amplify *mScarlet-I* with an overlapping sequence of 12-bp and 17-bp mismatches
M172	CCCGCGCGTTGGTAAGCGGATGCCGGG	
M201	GATCGGTGCGGGTGAGCGCAACGCAATT	To amplify *mScarlet-I* with an overlapping sequence of 12-bp and 20-bp mismatches
M202	CTCCCCGCGCGTGTAAGCGGATGCCGGG	
M251	AGGGCGATCGGTGCGCTCGAGCGCAACGCAATT	To amplify *mScarlet-I* with an overlapping sequence of 12-bp and 25-bp mismatches
M252	AACCGCCTCTCCCCGAATTCGGATGCCGGGAGCA	

M521	TTCGCCATTCAGGCTCGAGCGCAACGCAATT	To amplify *mScarlet-I* with an overlapping sequence of 12-bp and 52-bp mismatches
M522	TTGGCAAGCTGCTCTGAATTCGGATGCCGGGAGCA	
M100F	AAGGCGGGAAACTCGAGCGCAACGCA	To amplify *mScarlet-I* with an overlapping sequence of 12-bp and 52 base / 100 base mismatches with M522
2Frag1F	TCGCTATTACGCCAGCTCGAGCACTCAGGGCGCAAG	Two/Three fragments cloning.
2Frag1R	CAACCGAGCGTTCTGAAC	To amplify *sGFP2* with an overlap sequence of 15-bp
2Frag2F	CAGAACGCTCGGTTGGGAAGCCCTGC	Two/Three fragments cloning.
2Frag23R	GATTCATTAATGCAGATGGAGTTCTGAGGTCATT	To amplify *mScarlet-I* with an overlapping sequence of 15-bp
3Frg31	CAGAACGCTCGGTTGCAGTGAGCGCAACGC	Three fragments cloning.
3Frg32	CGGGAGCAGACAAGCC	To amplify *LacZα* with an overlap sequence of 15-bp
3Frg33	GCTTGTCTGCTCCCGGTTGGGAAGCCCTGC	Three fragments cloning. To amplify *mScarlet-I* with an overlapping sequence of 15-bp
1305RF	CCTGTGGTTGGCATGCA	Sanger Sequencing primer
DsRedF	TCCCACAACGAGGACTACAC	Sanger Sequencing primer

Supplementary Table S2. Number of clones obtained from DBR cloning under different treatment temperatures

	25℃		40℃		55℃		65℃	
	Number of total colonies	Number of red fluorescent colonies	Number of total colonies	Number of red fluorescent colonies	Number of total colonies	Number of red fluorescent colonies	Number of total colonies	Number of red fluorescent colonies
Replicate 1	324	144	1412	1200	2508	2088	1776	1420
Replicate 2	340	136	1304	980	2284	1920	1792	1364
Replicate 3	352	140	1360	1040	2184	1856	1500	1200
average	338.7	140	1358.7	1073.3	2325.3	1954.7	1689.3	1328

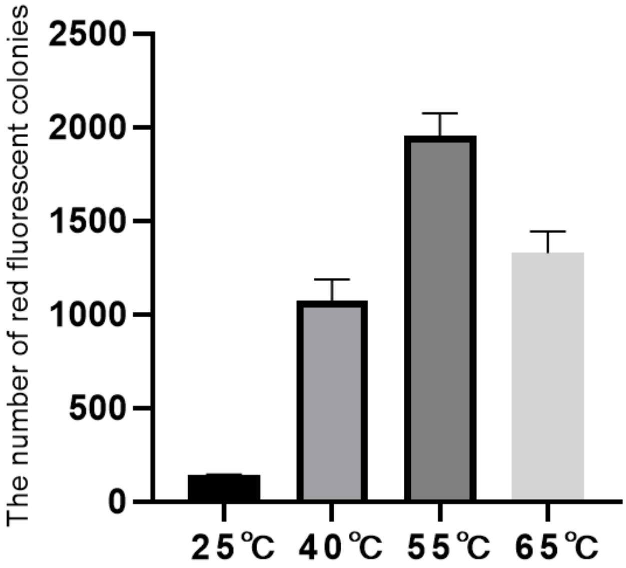

Supplementary Figure S1. Comparison of the number of positive clones obtained from DBR Cloning under different treatment temperatures. Results are the mean ± SD for three biological replicates.

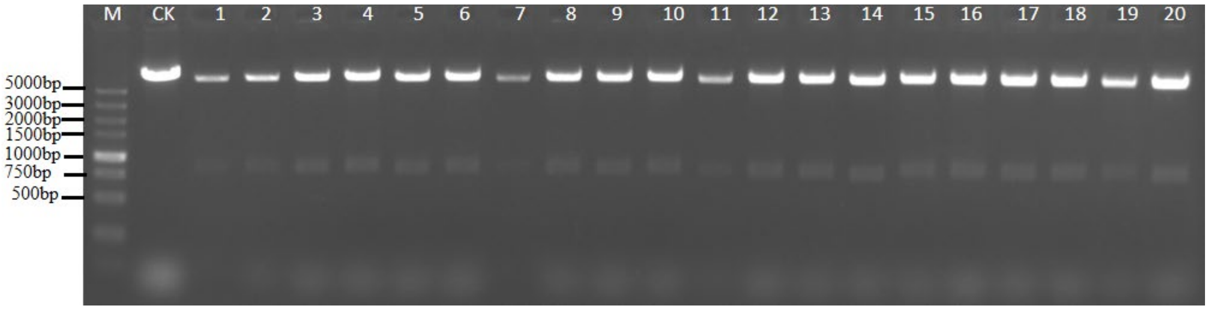

Supplementary Figure S2. Electrophoresis profile of L7-18 and M11 were cloned into vector pCAMBIA-1305.1 followed by *Hin*dIII restriction enzyme digestion verification. Lane 1-10: L7-18 transformants emitting red fluorescence. Lane 11-20: M11 transformants emitting red fluorescence. Lane CK: the vector pCAMBIA-1305.1. Lane M: DL5000 marker (Sangon Biotech (Shanghai) Co., Ltd. China).

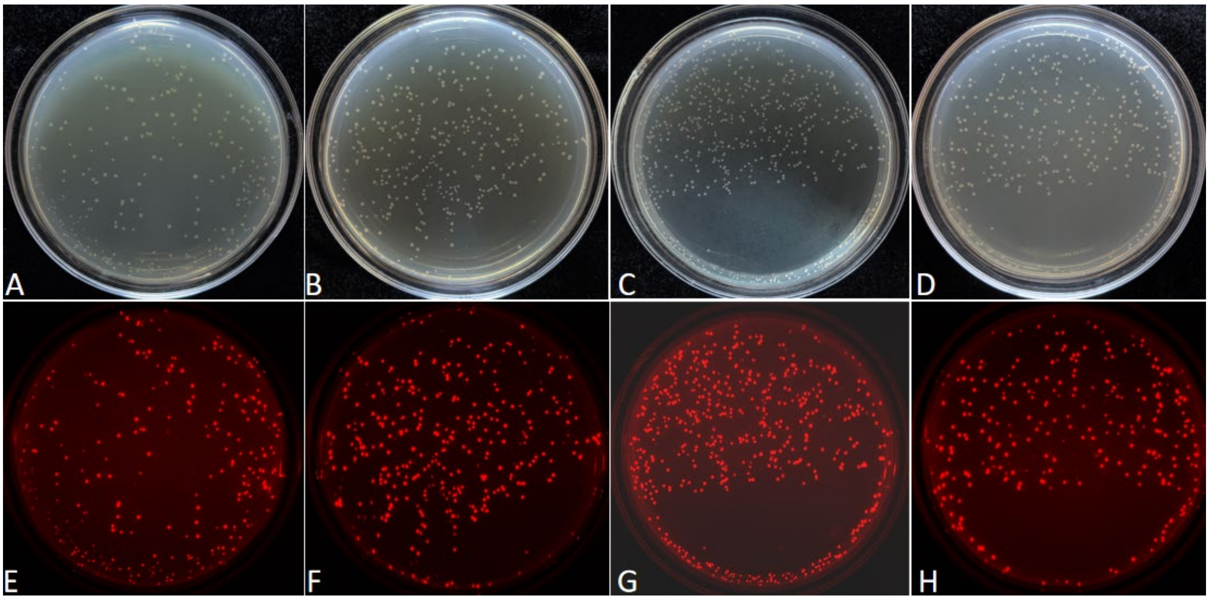

Supplementary Figure S3. Examples of transformation plates of the FDFs with different overlapping sequence. A quarter of the transformed bacterial solution was spread on one LB/Kan plate. A/E, B/F, C/G, and D/H showed the transformation recombinants with 14, 16, 18, and 20-bp, respectively. A-D plates were pictured in natural light and E-H were detected with excitation at 540 nm, emission at 600 nm.

Supplementary Table S3. The total number of colonies and the number of colonies emitting red fluorescence were counted after the FDFs with different overlapping sequences were mixed with the vector and subjected to temperature treatment at a maximum temperature of 55℃ via HBR cloning,

	8-bp		10-bp		12-bp		14-bp		16-bp		18-bp		20-bp	
	Number of total colonies	Number of red fluorescent colonies	Number of total colonies	Number of red fluorescent colonies	Number of total colonies	Number of red fluorescent colonies	Number of total colonies	Number of red fluorescent colonies	Number of total colonies	Number of red fluorescen t colonies	Number of total colonies	Number of red fluorescent colonies	Number of total colonies	Number of red fluorescen t colonies
Replicate 1	485	216	740	480	1280	928	1660	1326	2256	1940	2456	2104	1488	1012
Replicate 2	427	176	726	500	1560	1124	1440	1180	2116	1840	2344	1968	1416	920
Replicate 3	420	196	761	460	1368	1026	1704	1396	1888	1624	2260	1900	1384	968
Average	444	196	742.3	480	1402.7	1026	1601.3	1300.7	2086.7	1801.3	2353.3	1990.7	1429.3	966.7

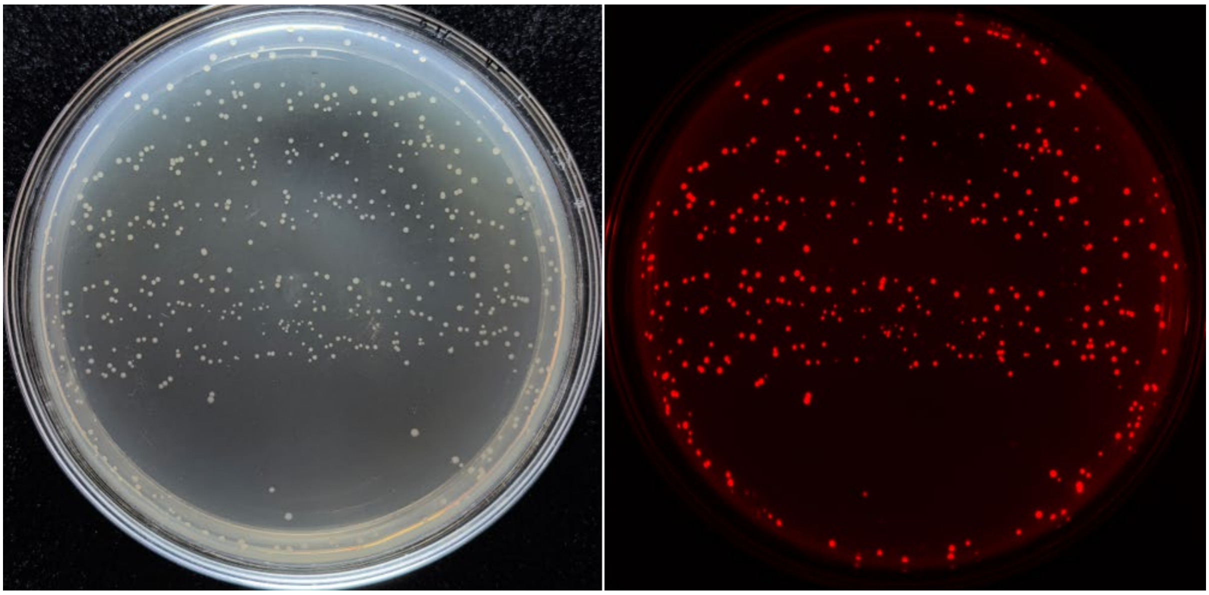

Supplementary Figure S4. The transformation plate of FDF with 20-bp overlapping sequence and the vector treated with the maximum temperature of 65℃. The left plate was pictured in natural light and the rihgt plate was detected with excitation at 540 nm, emission at 600 nm. A quarter of the transformed bacterial solution was spread on one LB/Kan plate.

Supplementary Table S4. Number of clones obtained from DBR cloning of FDF fragments carrying different mismatched bases at a treatment temperature of 55 °C

	M5		M7		M9		M11	
	Number of total colonies	Number of red fluorescent colonies	Number of total colonies	Number of red fluorescent colonies	Number of total colonies	Number of red fluorescent colonies	Number of total colonies	Number of red fluorescent colonies
Replicate 1	708	404	692	356	560	340	368	232
Replicate 2	660	408	580	376	512	288	412	204
Replicate 3	756	448	524	320	544	304	400	192
average	708	420	598.67	350.67	538.67	310.67	393.33	209.33
The percentage of positive clones		59.32%		58.57%		57.67%		53.22%

A

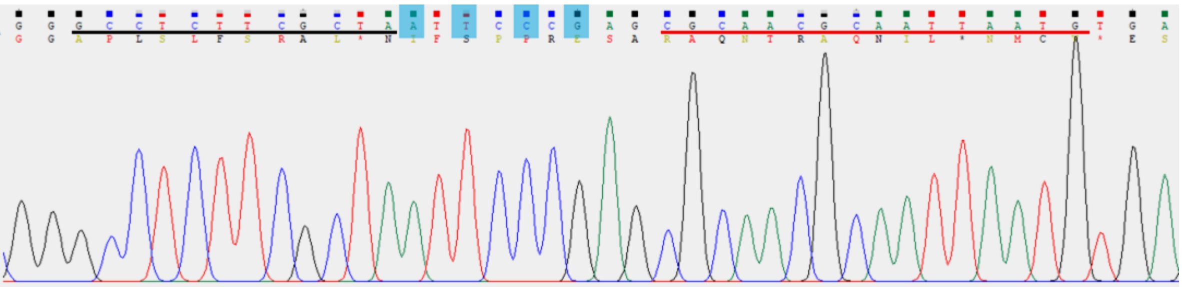

The two blunt-end sequences of pCAMBIA-1305.1 digested with *Pvu*II (CAG ↓CTG):

---TGCGGGCCTCTTCGCTATTACGCCAG-3’ ↓ 5’-CTGCATTAATGAATCGGCCAA---

M901 forward primer: 5’-GCCTCTTCGCTAATTCCCGAGCGCAACGCAATTAATG-3’

B

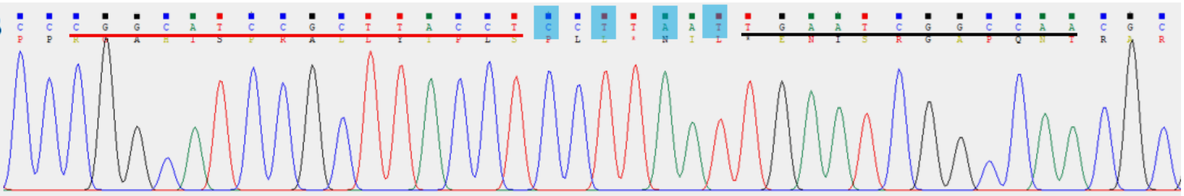

The two blunt-end sequences of pCAMBIA-1305.1 digested with *Pvu*II (CAGCTG):

---TGCGGGCCTCTTCGCTATTACGCCAG-3’ ↓ 5’-CTGCATTAATGAATCGGCCAA---

M902 reverse primer: 5’-TTGGCCGATTCAATTAAGGAGGTAAGCGGATGCCG-3’

Reverse Complimentary sequence of M902:5’-CGGCATCCGCTTACCTCCTTAATTGAATCGGCCAA-3’

C

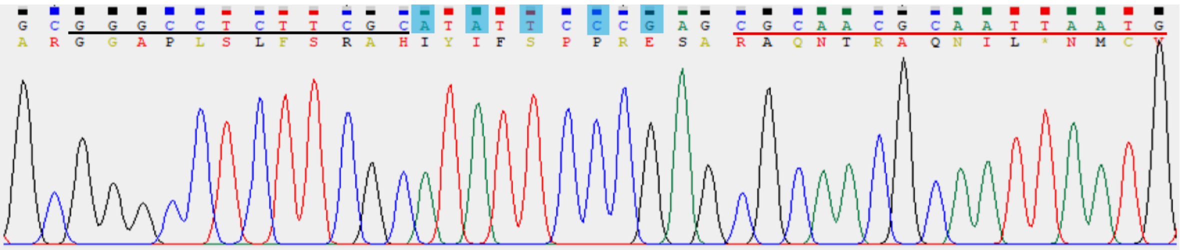

The two blunt-end sequences of pCAMBIA-1305.1 digested with *Pvu*II:

---TGCGGGCCTCTTCGCTATTACGCCAG-3’ ↓ 5’-CTGCATTAATGAATCGGCCAA---

M111 forward primer: 5’-GGGCCTCTTCGCATATTCCCGAGCGCAACGCAATTAATG-3’

D

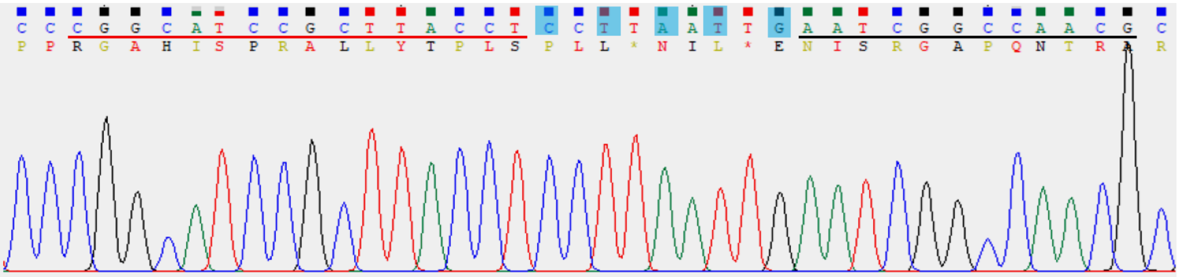

The two blunt-end sequences of pCAMBIA-1305.1 digested with *Pvu*II (CAG ↓CTG):

---TGCGGGCCTCTTCGCTATTACGCCAG-3’ ↓ 5’-CTGCATTAATGAATCGGCCAACG---

M112 reverse primer: 5’-CGTTGGCCGATTCAATTAAGGAGGTAAGCGGATGCCG-3’

Reverse Complimentary sequence of M112:5’-CGGCATCCGCTTACCTCCTTAATTCAATCGGCCAACG-3’

Supplementary Figure S5. The Sanger sequencing of M9 and M11 positive transformants.The red underlined sequence is the primer that perfectly matches the template for PCR amplification. The black underlined sequence is the overlapping sequence with the vector. The blue-color highlighted bases are the mismatched bases.

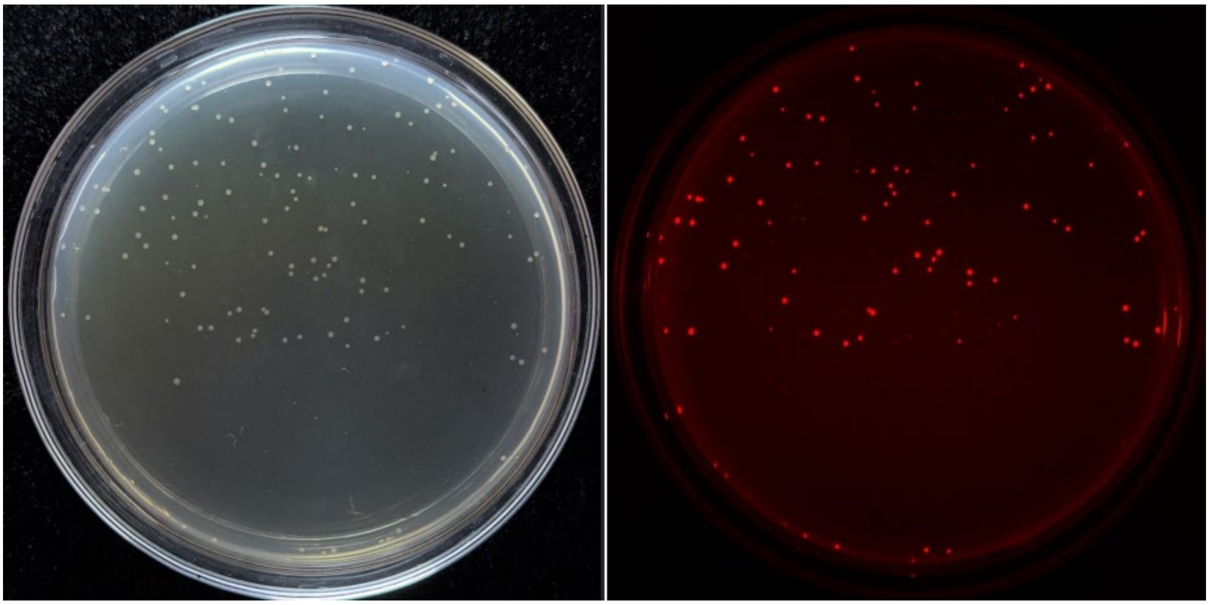

Supplementary Figure S6 M11 FDF was cloned into pCAMBIA-1305.1 with HBR with 70°C. A quarter of the transformed bacterial solution was spread on one LB/Kan plate. The left plate was pictured in natural light and the rihgt plate was detected with excitation at 540 nm, emission at 600 nm.

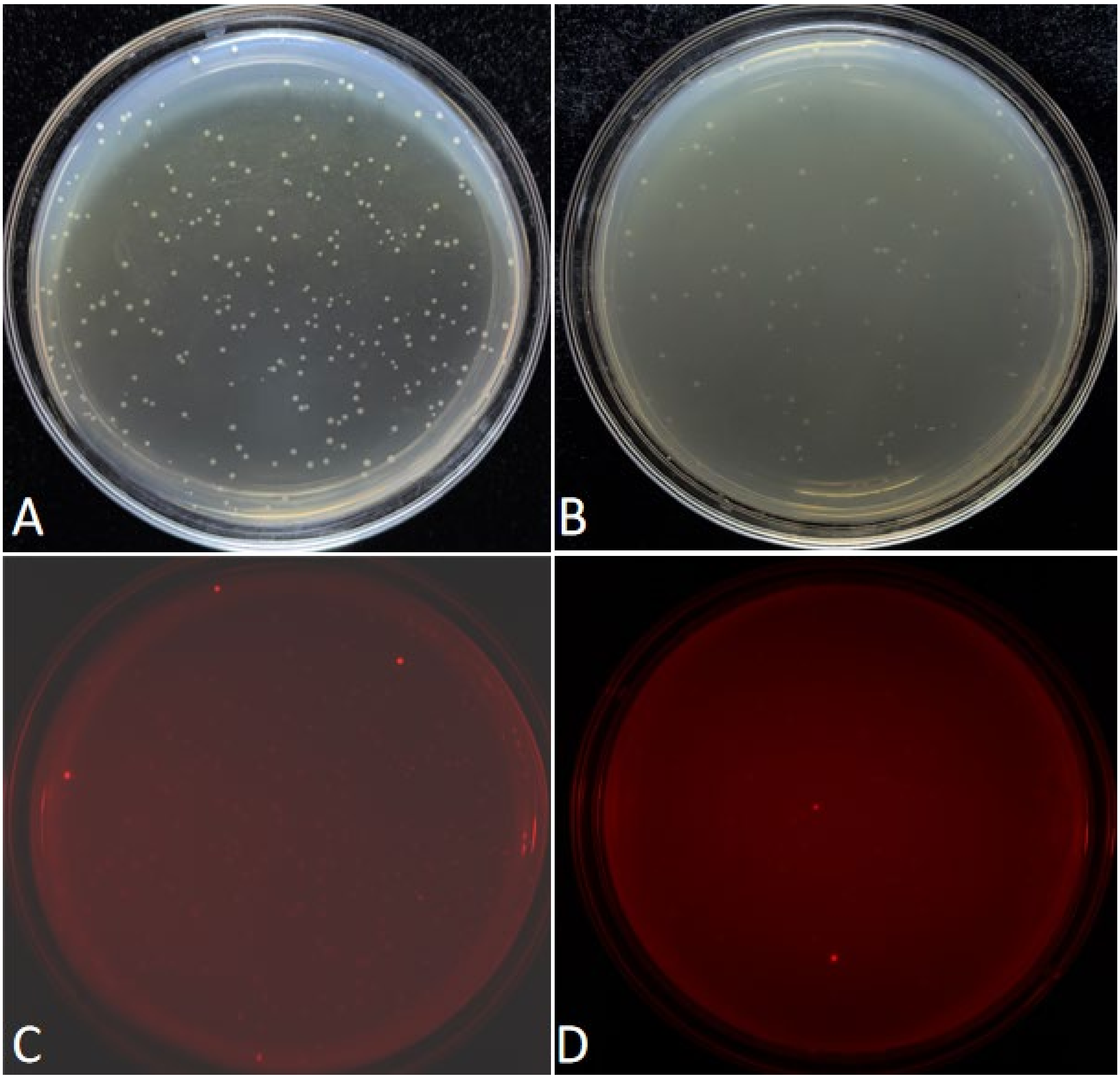

Supplementary Figure S7. M521/M522 (A/C), and M100F/M522 (B/D) were cloned into pCAMBIA-1305.1 with HBR. A/B were pictured in natural light and C/D were detected with excitation at 540 nm, emission at 600 nm. A quarter of the transformed bacterial solution was spread on one LB/Kan plate.

Supplementary Table S5. Total number of clones / number of positive clones obtained by DBR cloning of FDF fragments carrying different mismatched bases.

	M17		M20		M25		M52		M100	
	Number of total colonies	Number of red fluorescent colonies	Number of total colonies	Number of red fluorescent colonies	Number of total colonies	Number of red fluorescent colonies	Number of total colonies	Number of red fluorescent colonies	Number of total colonies	Number of red fluorescent colonies
Replicate 1	1748	244	1156	184	1052	20	304	16	380	8
Replicate 2	1424	184	956	148	820	20	284	8	324	4
Replicate 3	1160	152	924	130	712	16	292	0	196	0
Average	1444	193.33	1012	154	861.3	18.67	293.33	8	225	4
The percentage of positive clones		13.39%		15.2%		2.17%		2.73%		1.78%

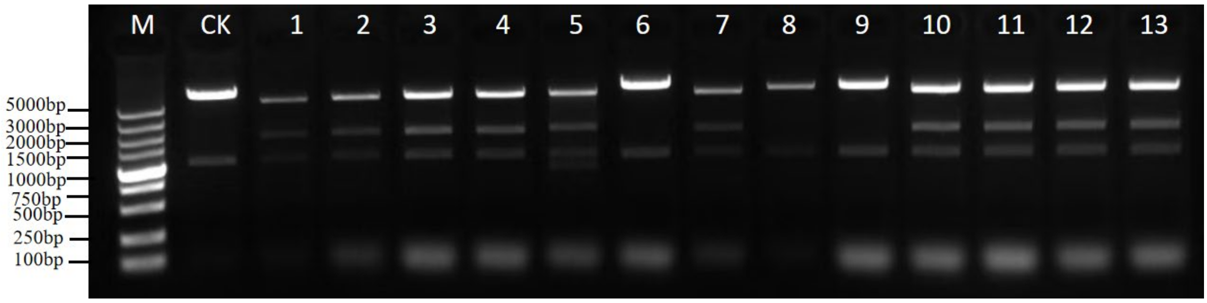

Supplementary Figure S8 Electrophoresis profile of M25 and M52 were cloned into vector pCAMBIA-1305.1 followed by *Xho*I restriction enzyme digestion verification. Lane M: DL5000 marker (Sangon Biotech (Shanghai) Co., Ltd. China), Lane CK: the vector pCAMBIA-1305.1, Lane 1-6: six clones emitting red fluorescence from M25, Lane 7-13: seven clones emitting red fluorescence from M52.

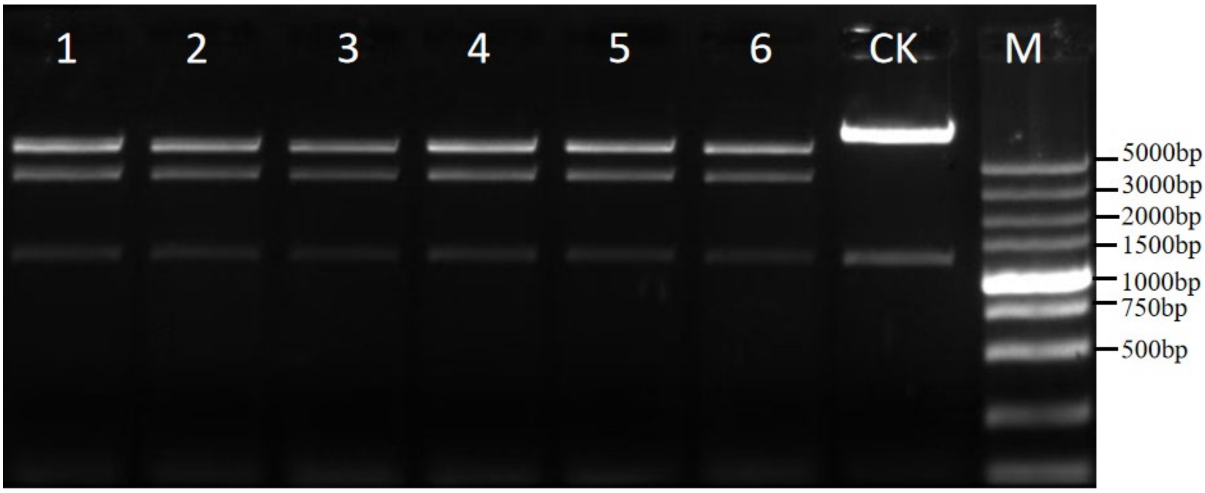

Supplementary Figure S9 Electrophoresis profile of three FDFs were cloned into vector pCAMBIA-1305.1 followed by *Xho*I restriction enzyme digestion verification. Lane 1-6: six clones emitting red fluorescence. Lane CK: the vector pCAMBIA-1305.1. Lane M: DL5000 marker (Sangon Biotech (Shanghai) Co., Ltd. China).

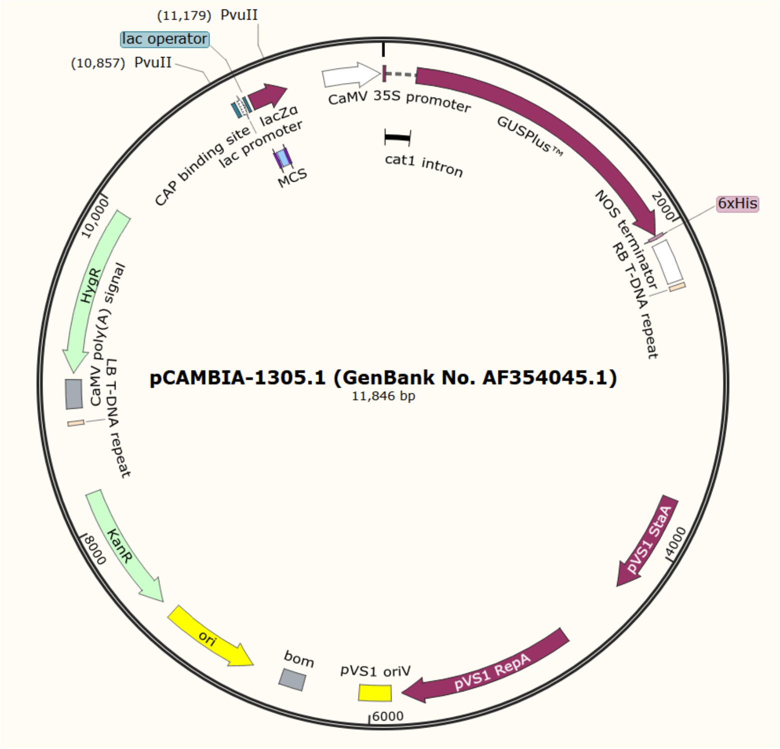

Supplementary Figure S10 Diagrammatic presentation of the pCAMBIA1305.1. The cloning site is *Pvu*Ⅱ.
